# Anti-Allergic Inflammatory Effect of *Agarum cribrosum* and Its Phlorotannin Component, Trifuhalol A, against the Ovalbumin-Induced Allergic Asthma Model

**DOI:** 10.3390/cimb45110557

**Published:** 2023-11-05

**Authors:** Joonki Kim, Sang Heon Lee, Siqi Zhang, Sim-Kyu Bong, Aaron Taehwan Kim, Hara Lee, Xiaoyong Liu, Sang Moo Kim, Su-Nam Kim

**Affiliations:** 1 Natural Products Research Center, Korea Institute of Science and Technology (KIST), Gangneung 25451, Republic of Korea; need2pro@kist.re.kr (J.K.); 120054@kist.re.kr (S.H.L.); 120060@kist.re.kr (S.Z.); bsk0125@korea.kr (S.-K.B.); 122051@kist.re.kr (H.L.); 2 Division of Bio-Medical Science and Technology, KIST School, University of Science and Technology, Seoul 02792, Republic of Korea; 3 Department of Food Science, University of Massachusetts, Amherst, MA 01003, USA; aaronkim@umass.edu; 4 Department of Dentistry, Gangneung-Wonju National University, Gangneung 25457, Republic of Korea; 5 Haizhibao Deutschland GmbH, Heiliggeistgasse, 85354 Freising, Germany; xyliu@chengshan.com; 6 Department of Marine Food Science and Technology, Gangneung-Wonju National University, Gangneung 25457, Republic of Korea; smkim@gwnu.ac.kr

**Keywords:** *Agarum cribrosum*, trifuhalol A, asthma, allergic, ovalbumin, inflammation

## Abstract

Asthma is a chronic inflammatory disease involving structural changes to the respiratory system and severe immune responses mediated by allergic cytokines and pro-inflammatory mediators. *Agarum cribrosum* (AC) is a kind of seaweed which contains a phlorotannin, trifuhalol A. To evaluate its anti-allergic inflammatory effect against asthma, an ovalbumin inhalation-induced mouse asthma model was used. Histologic observations proved that trifuhalol A is minimizing the lung and tracheal structure changes as well as the infiltration of eosinophils and mast cells against ovalbumin inhalation challenge. From the serum and bronchoalveolar lavage fluid, ovalbumin-specific IgE and Th2-specific cytokines, IL-4, -5, and -13, were reduced with trifuhalol A treatment. In addition, IL-1β, IL-6, and TNF-α concentrations in lung homogenate were also significantly reduced via trifuhalol A treatment. Taken together, trifuhalol A, isolated from AC, was able to protect lung and airways from Th2-specific cytokine release, and IgE mediated allergic inflammation as well as the attenuation of IL-1β, IL-6, and TNF-α in lung, which results in the suppression of eosinophils and the mast cells involved asthmatic pathology.

## 1. Introduction

Asthma is a chronic inflammatory disease characterized by structural changes including airway wall remodeling and smooth muscle increment in addition to a cytokine response dominated by Th2 cytokines. The interaction of the respiratory system with allergen, pollutant, and virus exposure are known to be among the important causes of asthma. Even though recent worldwide surveillance reports a decreasing trend of prevalence and mortality of asthma patients, close to 300 million clinical cases are being estimated, and over 450 thousand death cases have been reported [1]. In particular, the economic burden due to asthma is continuously rising worldwide.

Allergic asthma, associated with the exposure to aero-allergens including house dust mite, pollens, fungi, and animal dander, is the most common type of asthma [2]. The recurrent exposure of the respiratory system to such allergens leads to the activation of antigen-presenting cells. Eventually, the T-helper cell population expands and becomes hyperactivated to secrete cytokines and promote the infiltration of various immune cells in the lung and airway to subsequently release the pro-inflammatory factors [3]. While the Th2-specific immune response is being introduced as a major allergic inflammation pathway, the involvement of Th1/Th17 responses is continuously being reported to increase the complexity of inflammation found in asthmatic patients [4].

Despite the moderately successful utilization of anti-inflammatory steroid therapy, the complexity of immune responses and multi-cell type inflammations is being correlated with increasing clinical reports of drug-resistant asthmatic cases [5,6]. Moreover, symptomatic relief is insufficient with chronic cases of asthma, and younger age patients are often exposed to the severe side effects of steroid therapeutics. Phytochemicals isolated from natural products have always been a popular choice for drug candidates in particular, targeting chronic diseases with complex pathophysiologic mechanisms. Considering the nature of asthma therapeutics, research on phytochemicals that can be administered for prolonged periods with a wider safety margin will be given with priority. *Agarum cribrosum* (AC), also named as *Agarum clathratum*, is a common seaweed that can be found at the east coast of Korea, Hokkaido in Japan, Kuril Islands, Bering sea, and the shores of American Pacific Ocean. There are a limited number of previous reports on the effect of various types of AC extracts, but since 2000, antioxidative activity [7,8], treatment of constipation and reducing blood lipids concentration [9,10], protecting epidermal keratinocytes against UVA-irradiation [11], an anti-cancer effect on ovarian cancer cell lines [12], and a neuroprotective effect on transient cerebral ischemia model [13] have been reported. In 2018, the authors reported a novel method of phlorotannin extraction from AC and profiled the existence of trifuhalol A within the AC extract [14]. Since then, a number of reports have indicated the antioxidative and antidiabetic activities [15] and tyrosinase-inhibitory activity [16] of trifuhalol A. In addition, authors have recently reported the anti-inflammatory activity of trifuhalol A against LPS-stimulation and atopic dermatitis [17,18]. However, until now, the application of AC and trifuhalol A on respiratory diseases has not been researched. Considering the previous reports on the pharmaceutical effects of AC and trifuhalol A on various diseases-related models, the development of medical agents using AC and trifuhalol A is promising. Moreover, compared to the phytochemicals from inland plant resources, oceanic plants have been reported for a more limited area of diseases and resources. In the current study, the authors report for the first time the anti-allergic inflammation effect of AC extract and its phlorotannin component, trifuhalol A, on a in vivo model of allergic asthma, which can lead to the development of a medication candidate from marine resources.

## 2. Materials and Methods

### 2.1. Preparation of A. cribrosum Extract and Trifuhalol A

The 70% acetone extract of AC and isolated trifuhalol A, a phlorotannin, was provided by Prof. Sang Moo Kim (Gangneung-Wonju National University, Gangneung, Republic of Korea). AC was collected from the coast of Gangneung, Republic of Korea, in 2015. The collected AC samples were dried and grounded to pass through a 300 μm sieve. The AC powder was extracted with 70% acetone using ultrasound at a frequency of 35 kHz and an intensity of 56.58 W/cm^2^ for 5.75 h at 30 °C, as previously reported by Phasanasophon and Kim [14]. The extract was divided into multiple fractions and purified to isolate phlorotannin, and trifuhalol A was identified using ^1^H-NMR, ^13^C-NMR, and liquid chromatography–mass spectroscopy analyses [14,17]. The calculated total yield of isolated trifuhalol A was 0.85% from the extract utilizing 100 g of dried AC. The specimen of AC is kept in the herbarium of Gangneung-Wonju National University, and the detailed chemical profile data of AC extract were previously reported by the authors [14].

### 2.2. Animal Treatments and Induction of Allergic Asthma In Vivo Model

Seven-week-old female BALB/c mice were purchased from Orient Bio (Seongnam, Republic of Korea) and housed under specific pathogen-free conditions at 24 ± 2 °C, 50 ± 15% humidity and a 12-h light–dark cycle. All procedures involving animals were approved by the Korea Institute of Science and Technology Animal Care Committee (approval No. KIST-2021-06-067) and performed in accordance with ethical guidelines. After a week of acclimatization, mice were divided into six experimental groups (N = 6 per group): normal animal group, ovalbumin ((OVA) Sigma-Aldrich, CA, USA)-induced asthma group, OVA with dexamethasone (Sigma-Aldrich) 1.5 mg/kg treatment group, OVA with AC extract 100 mg/kg treatment group, and OVA with trifuhalol A 10 and 20 mg/kg treatment groups. All OVA-treated groups were sensitized with intraperitoneal (i.p.) injection of OVA (1 mg/kg) along with aluminum hydroxide (200 mg/mL saline) on day 1 and 8. Then, all mice except for the normal group was challenged for 30 min/day with 5% OVA (*w/v*) solution, aerosolized by the ultrasonic nebulizer from day 15 for 4 days. AC extract and trifuhalol A were dissolved in 0.5% carboxymethyl cellulose solution and administered orally every day from day 1. For positive control, dexamethasone was i.p. injected 30 min before daily OVA inhalation challenges at the concentration of 1.5 mg/kg. On day 19, all animals were sacrificed to collect samples for analyses. Blood was collected and centrifuged to separate serum samples, and spleen was collected to measure the organ weight. Bronchoalveolar lavage was repeated three times with 3 mL of cold sterile saline and then centrifuged to collect the bronchoalveolar lavage fluid (BALF). Right lung tissues were collected and immediately frozen for protein extraction. Left lung and trachea tissues were collected and fixed in 10% neutral-buffered formalin solution for histologic analysis. All samples, except for the lung tissue for histology, were kept frozen at −80 °C until further analyses.

### 2.3. Histological Examination

Formalin-fixed lung and trachea tissues were processed for paraffin wax embedding. The embedded tissues were sectioned in 2–3 mm thickness. Hematoxylin and eosin (H&E) staining was performed on lung tissue sections, and toluidine blue staining was performed on trachea sections to measure the epithelium thickness and inflammatory cell migrations. In particular, the migration of mast cells was counted via toluidine blue-stained slides targeting dark blue-to-purple color-stained cells within the region of interest. The stained tissue slides were analyzed using an optical microscope (Olympus CX31/BX51; Olympus Optical Co., Tokyo, Japan).

### 2.4. Lung Tissue Homogenate Preparation

Frozen lung tissues were homogenized using PRO-PREP (iNtRON Biotechnology, Seongnam, Republic of Korea) solution containing a protease inhibitor cocktail based on the 1:1 (*w/v*) tissue/solution ratio at 4 °C. Lung homogenate samples were centrifuged at 9000× *g* for 10 min at 4 °C to collect the protein-containing supernatant. Total protein concentration of acquired lung homogenate were determined using BCA kit (Thermo Fisher Scientific, Waltham, MA, USA).

### 2.5. Measurement of Immunoglobulin and Inflammatory Cytokines

The enzyme-linked immunosorbent assay (ELISA) kits were used to measure the levels of mouse OVA-specific immunoglobulin E (IgE; Cat. No. 439807, Biolegend, San Diego, CA, USA), interleukins (IL)-1β (Cat. No. ab197742, Abcam, Cambridge, UK), -4, -5, -13 (Cat. No. M4000B-1, M5000, DY413-05, R&D Systems, Minneapolis, MN, USA), and tumor necrosis factor-alpha (TNF-α) (Cat. No. 88-7324-88, Thermo Fisher Scientific) from serum, BALF, and lung homogenate samples based on the manufacturer’s instructions. The resulting absorbance was measured at 450 nm with a microplate reader.

### 2.6. Statistical Analysis

All results are presented as the mean ± standard deviation (S.D.). The statistical significance of differences among the experimental groups was analyzed via one-way analysis of variance (ANOVA) with Prism software (GraphPad Software Inc.; Boston, MA, USA https://www.graphpad.com/features, accessed on 17 October 2023), and Tukey’s honest significant difference test was used as the post hoc test. *p*-values under 0.05 were considered statistically significant.

## 3. Results

### 3.1. AC Extract and Trifuhalol A Reduce Histologic Changes in Lung and Trachea

**Figure 1:**
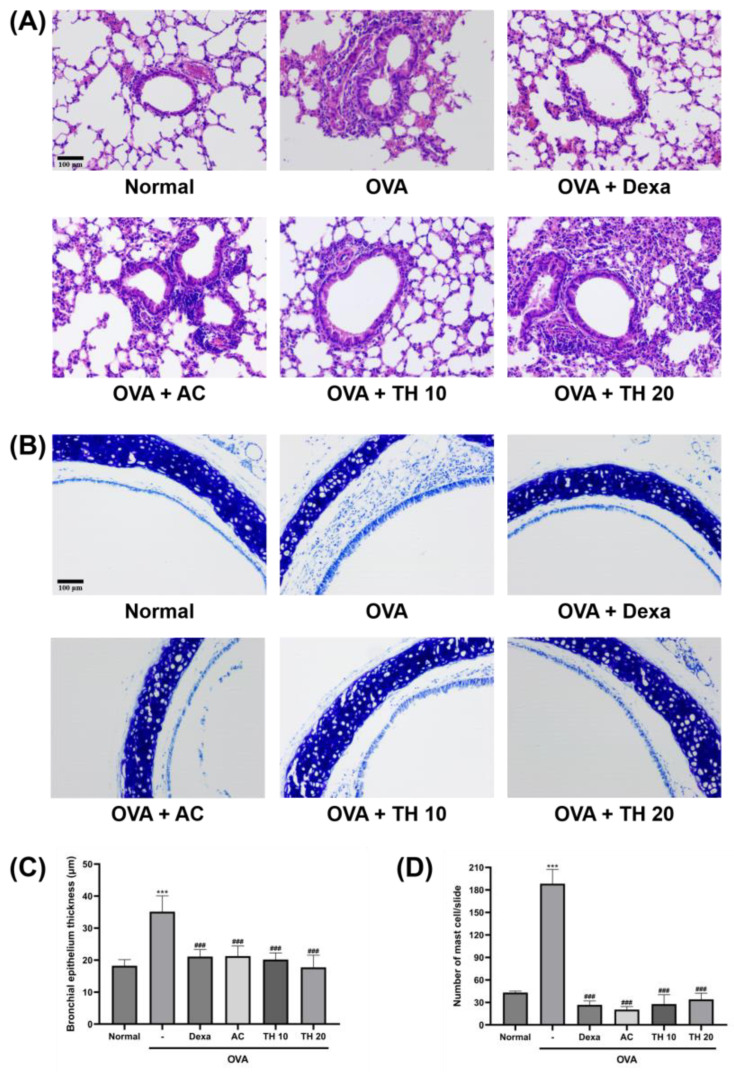
Effect of AC extract and trifuhalol A on histological changes of lung and trachea exposed to OVA inhalation. (**A**) Representative H&E-stained sections of lung tissues focused on bronchiole. (**B**) Representative toluidine blue-stained sections of trachea. (**C**) Average measurements of bronchial epithelium thickness. (**D**) Average number of mast cells infiltrated into submucosa region and respiratory epithelium of trachea. Dexamethasone (Dexa) 1.5 mg/kg (i.p.), AC extract 100 mg/kg (p.o.), Trifuhalol A (TH) 10, and 20 mg/kg (p.o.) were treated for each group. Original magnification × 200. Data are presented as mean ± S.D. (N = 5/group). *** Significantly different (*p* < 0.001) from the normal group. ^###^ Significantly different (*p* < 0.001) from the OVA inhalation group.

To visualize the structural changes and determine the effect of AC extract and trifuhalol A on lung and trachea, histologic analyses were employed (Figure 1). H&E-stained lung tissues showed intact bronchial epithelium, with no sign of immune cell infiltration (Figure 1A). After OVA inhalation challenges, the lung of mice exhibited significantly thickened bronchial epithelium (35.1 ± 4.5 μm), with numerous signs of eosinophil infiltration (Figure 1C). AC extract 100 mg/kg treatment significantly reduced the bronchial epithelium thickening down to 21.2 ± 2.9 μm, which is a similar level to the average of the dexamethasone-treated group (21.1 ± 2.0 μm). Both trifuhalol A 10 and 20 mg/kg treatment also significantly attenuated this trend in a dose-dependent manner (20.1 ± 1.9 and 17.7 ± 3.5 μm, respectively). The horizontal sections of the trachea, with toluidine blue staining, clearly showed a thickening trend of respiratory epithelium induced via exposure to OVA inhalation (Figure 1B). The infiltration of mast cells into the expended submucosa region was significant in the OVA-exposed group (188.3 ± 15.5 cells/slide), and AC extract treatment significantly reduced the infiltration (20.3 ± 3.4 cells/slide) similar to the normal range, as well as the dexamethasone-treated group (26.7 ± 4.2 cells/slide) (Figure 1D). The trifuhalol A 10- and 20 mg/kg-treated groups also showed a significant reduction of mast cell infiltration (27.7 ± 10.2 and 34.0 ± 6.7 cells/slide, respectively).

### 3.2. AC Extract and Trifuhalol A Reduce Levels of OVA-Specific IgE and Allergic Cytokines in Both Serum and BALF

**Figure 2:**
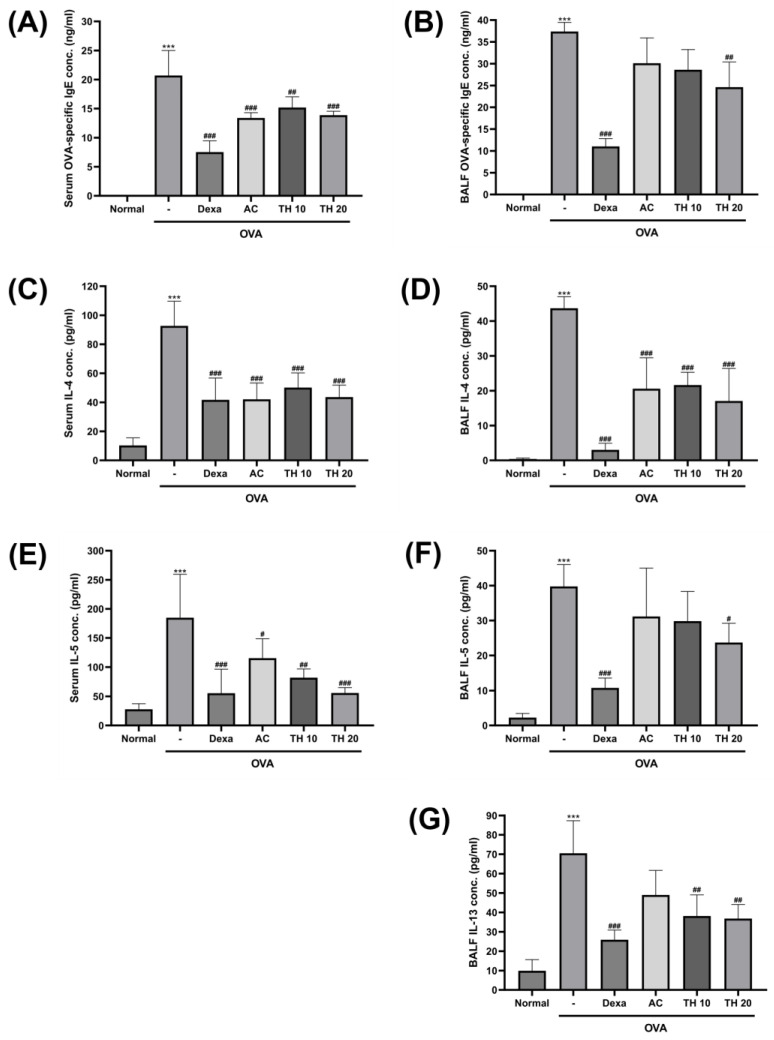
Effect of AC extract and trifuhalol A on OVA-specific IgE and allergic cytokines concentrations in serum and BALF after exposure to OVA inhalation. Measurement of average serum—(**A**) OVA-specific IgE, (**C**) IL-4, and (**E**) IL-5—and BALF—(**B**) OVA-specific IgE, (**D**) IL-4, (**F**) IL-5, (**G**) IL-13—concentrations using ELISA. Dexamethasone (Dexa) 1.5 mg/kg (i.p.), AC extract 100 mg/kg (p.o.), and Trifuhalol A (TH) 10 and 20 mg/kg (p.o.) were treated for each group. Data are presented as mean ± S.D. (N = 5/group). *** Significantly different (*p* < 0.001) from the normal group. ^#,##,###^ Significantly different (*p* < 0.05, *p* < 0.01, *p* < 0.001) from the OVA inhalation group.

To investigate the magnitude of allergic response induced via OVA inhalation exposure, the level of OVA-specific IgE and relevant cytokines, IL-4 and -5, were measured from the serum samples (Figure 2A,C,E). The OVA-specific IgE was undetectable in the normal group; however, OVA inhalation significantly increased it by 20.7 ± 3.9 ng/mL (Figure 2A). While the dexamethasone-treated group showed the most significantly reduced level of OVA-specific IgE (7.5 ± 1.7 ng/mL), the AC extract 100 mg/kg, trifuhalol A 10, and 20 mg/kg treatment groups all showed significant attenuation of OVA-specific IgE level (13.4 ± 0.8, 15.2 ± 1.7, and 13.9 ± 0.6 ng/mL, respectively). Serum levels of IL-4 and -5 were both significantly spiked with OVA inhalation (92.6 ± 15.2 and 184.8 ± 66.9 pg/mL, respectively), and the AC extract 100 mg/kg and trifuhalol A 10 and 20 mg/kg treatment groups significantly reduced the increased levels, along with the dexamethasone-treated group (Figure 2C,E). In particular, for the serum IL-4 level, AC extract 100 mg/kg (42.1 ± 10.1 pg/mL) and trifuhalol A 20 mg/kg treatment (43.5 ± 7.4 pg/mL) exhibited a significant reduction of serum IL-4 concentration, similar to the level of the dexamethasone-treated group (41.7 ± 13.5 pg/mL) (Figure 2C). AC extract 100 mg/kg treatment also significantly attenuated the increase in serum IL-5 down to 115.2 ± 30.7 pg/mL. Trifuhalol A 10 and 20 mg/kg treatment exhibited superior reduction of serum IL-5 compared to the AC extract-treated group in a dose-dependent manner (82.0 ± 13.1 and 55.8 ± 8.7 pg/mL, respectively) (Figure 2E).

Inflammatory cytokines secreted from the lung and airway directly affected by allergens, including OVA, can be efficiently detected from the BALF. The levels of OVA-specific IgE and relevant cytokines, IL-4, -5, and -13, were measured from the BALF samples (Figure 2B,D,F,G). The basal level of OVA-specific IgE was close to the minimum detectable range; however, OVA inhalation exposure significantly increased it up to 37.4 ± 1.8 ng/mL, which was higher than that observed in serum (Figure 2B). This increase in OVA-specific IgE level was most significantly decreased with dexamethasone 1.5 mg/kg treatment (11.0 ± 1.6 ng/mL); however, trifuhalol A 20 mg/kg treatment also showed a statistically significant level of decrement (24.6 ± 5.0 ng/mL). Even though statistical significance was not found, the AC extract 100 mg/kg and trifuhalol A 10 mg/kg treatment groups also showed a tendency to decrease the OVA-specific IgE level in BALF. OVA inhalation exposure also made the levels of BALF IL-4, -5, and -13 significantly increase (43.6 ± 2.9, 39.8 ± 5.4, and 70.5 ± 14.6 pg/mL, respectively) compared to the basal levels observed in the normal group (Figure 2D,F,G). AC extract 100 mg/kg and trifuhalol A 10 and 20 mg/kg treatments significantly attenuated the IL-4 levels in BALF (20.6 ± 7.2, 21.6 ± 3.0, and 17.0 ± 7.6 pg/mL, respectively) (Figure 2D). For the BALF IL-5 levels, only the dexamethasone 1.5 mg/kg and trifuhalol A 20 mg/kg treatment groups showed a significant reduction of IL-5 increment (10.8 ± 2.4 and 23.7 ± 4.8 pg/mL, respectively), while rest of the groups showed a decrementing tendency (Figure 2F). In the case of BALF IL-13 levels, which were not detected in serum samples, the AC extract 100 mg/kg treatment group showed a tendency towards IL-13 level attenuation; however, both of trifuhalol A 10 and 20 mg/kg treatments further attenuated the IL-13 level significantly (38.1 ± 9.5 and 36.8 ± 6.3 pg/mL, respectively) (Figure 2G).

### 3.3. Trifuhalol A Attenuate IL-1β, IL-6, and TNF-α Levels in the Lung after OVA Inhalation Exposure

**Figure 3:**
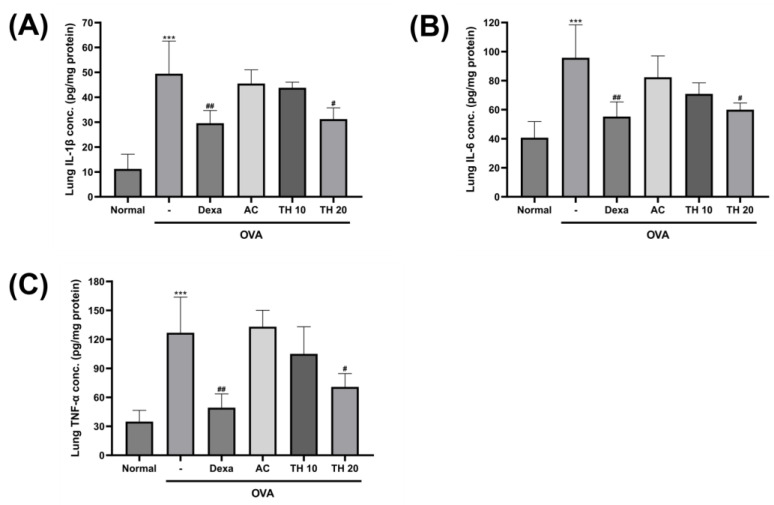
Effect of AC extract and trifuhalol A on lung tissue cytokines concentrations after exposure to OVA inhalation. Measurement of average lung homogenate (**A**) IL-1β, (**B**) IL-6, and (**C**) TNF-α concentrations using ELISA. Dexamethasone (Dexa) 1.5 mg/kg (i.p.), AC extract 100 mg/kg (p.o.), Trifuhalol A (TH) 10 and 20 mg/kg (p.o.) were treated for each group. Data are presented as mean ± S.D. (N = 5/group). *** Significantly different (*p* < 0.001) from the normal group. ^#,##^ Significantly different (*p* < 0.05, *p* < 0.01) from the OVA inhalation group.

IL1ꞵ, IL-6, and TNF-α are also considered as major mediators of lung and airway inflammatory responses induced by allergen exposures in experimental and clinical cases of asthma. Therefore, targeting these proteins in the development of asthma therapeutics is meaningful. From the lung tissue homogenate, the level of IL-1β was significantly upregulated after OVA inhalation exposure by over four times (49.4 ± 11.4 pg/mg protein) that of the normal group (11.2 ± 5.1 pg/mg protein) (Figure 3A). While dexamethasone 1.5 mg/kg treatment significantly suppressed this increment (29.5 ± 4.4 pg/mg protein), trifuhalol A 20 mg/kg treatment also showed significant suppression as well (31.2 ± 3.9 pg/mg protein). The levels of IL-6 and TNF-α in the lung tissue homogenate also revealed a similar trend. OVA inhalation exposure significantly increased both IL-6 and TNF-α levels (95.7 ± 19.7 and 126.8 ± 32.0 pg/mg protein, respectively) compared to the levels of the normal group (Figure 3B,C). While AC extract 100 mg/kg treatment showed an insignificant level of change, the trifuhalol A treatment groups exhibited a decreasing trend of IL-6 and TNF-α levels in lung homogenate, especially, with significance in the 20 mg/kg treatment group (60.0 ± 4.0 and 70.8 ± 11.9 pg/mg protein, respectively). Even though dexamethasone 1.5 mg/kg treatment showed a superior level of suppression for IL-6 and TNF-α levels (55.2 ± 8.8 and 49.3 ± 12.4 pg/mg protein, respectively) in lung homogenates, trifuhalol A 20 mg/kg treatment showed a competitive effect in the level of IL-6 suppression.

## 4. Discussion

Patients with asthma experience shortness of breathing, chest tightness or pain, wheezing, and coughing, which greatly affects daily life. Such symptoms are directly correlated with structural changes in the respiratory system, which are not limited to epithelial fragility, goblet cell hyperplasia, submucosal gland enlargement, and airway wall matrix deposition and thickening [19]. Such symptoms are well-observed on the experimental models of asthma, including allergic asthmatic mice induced via OVA exposure. As previously reported by multiple research groups, balb/c mice are especially sensitive to allergen responses and are the first choice in allergic animal studies [20,21]. There also exists a sex-dependent biological responsiveness to the allergens, and female balb/c mice are more preferred for experimental purpose. AC extract and trifuhalol A treatment effectively controlled the thickening of both lung and tracheal epithelium and the expansion of mucosal layers, which will eventually contribute to the relief of asthmatic symptoms.

In addition to structural changes, histologic analyses revealed OVA inhalation-induced eosinophils and mast cells’ infiltration into lung and trachea tissues. Eosinophils are a type of white blood cells which are involved in the inflammation of lung and airways in allergic asthma [22,23]. Eosinophils can promote the secretion of pro-inflammatory mediators, which are the major contributors to asthmatic inflammation, including airway epithelial damage, airway hyperresponsiveness, mucus hypersecretion, and airway remodeling [24,25,26]. Originating from the bone marrow as progenitor cells, the mast cells complete their differentiation in tissues in contact with the external environment, including respiratory tracts [27]. A variety of receptors expressed on mast cells enable the response to stimuli including OVA, and mast cells begin to release histamine and proteases, which contribute to asthmatic symptoms. AC extract and trifuhalol A treatment prominently reduced the infiltration of eosinophils and mast cells induced via OVA inhalation.

The involvement of immune cells, such as eosinophils and mast cells, during allergic asthma is well known to be triggered by multiple cytokines and immunoglobulin. When external allergen is presented to B cells, allergen-specific IgE is produced and secreted to mediate the immune responses. In our OVA inhalation model of allergic asthma, the OVA-specific IgE level spiked in both serum and BALF. AC extract and trifuhalol A treatment significantly suppressed the concentration of OVA-specific IgE found in serum, and trifuhalol A 20 mg/kg treatment was also significantly reduced it in BALF as well. The reduced concentration of OVA-specific IgE in serum and BALF will lead to the inhibition of mast cell degranulation and attenuate the release of numerous inflammatory factors that play roles in allergic asthma pathology [28]. The activation of eosinophils and mast cells is not only achieved via the involvement of allergen-specific IgE; various cytokines are also well established in their role in allergic asthma inflammation. Th2 cell-specific cytokines, including IL-4, -5, and -13, are the major players in the mechanism of allergic inflammation, and they have become major targets of asthma therapeutics. Both IL-4 and -13 act as major mediators in the eosinophil accumulation and IgE synthesis via B cells; thus, they contribute the mucus production, bronchial fibrosis, and airway hyperresponsiveness symptoms [29]. Moreover, mast cells are known to produce IL-4 and -13 in response to allergic pathway activation via IgE [30]. In serum and BALF, AC extract and trifuhalol A treatment were effective in decreasing the concentration of IL-4. For the IL-13, it was measured only from BALF, and while AC extract reduced the IL-13 concentration to an insignificant level, trifuhalol A treatment reduced the IL-13 to a significant concentration. IL-5 is a very selective cytokine to eosinophils and basophils due to the restricted expression of the receptor [31]. Considering the role of eosinophil in pulmonary inflammation, IL-5, which is involved in the maturation and release of eosinophil, will be one of the important targets in asthmatic inflammation. From the serum, IL-5 concentration was effectively reduced via AC extract and trifuhalol A treatments. In the BALF, however, the effect of the AC extract was weakened, and only trifuhalol A treatment proceeded to reduce the IL-5 concentration significantly.

Besides the controlling of Th2-specific cytokine pathways, there are other inflammatory cytokines which have been raised as targets for asthma treatment. Pro-inflammatory cytokine IL-1β is considered to be the gatekeeper of inflammation based on its ability to control multiple immune responses, including OVA-induced allergic asthma, which proved its necessity in producing another pro-inflammatory cytokine release [32,33]. Both IL-6 and TNF-α are clinically proven inflammatory cytokines found in various types of asthma induced by allergens [34,35]. In asthma, IL-6 can be produced by primary lung epithelial cells, where allergic stimulus initiates the allergic inflammatory response. IL-6 was reported to promote IL-4 production during Th2 cell differentiation and inhibit Th1 differentiation, which caused IL-6 to be highlighted as a major potential contributor of the allergic asthma pathology [36,37]. TNF-α is another inflammatory mediator involved in various kinds of diseases. With the finding of TNF-α in asthmatic patients, the correlation of asthmatic symptoms and TNF-α has been widely studied [38,39]. TNF-α is also known to induce histamine release from mast cells and to be involved in mast cell–smooth muscle interaction, contributing to airway hyperresponsiveness in asthma [34,40]. In the current study, trifuhalol A treatment, not that of AC extract, showed a significant effect in reducing the levels of IL-1β, IL-6, and TNF-α in mouse lungs exposed to OVA inhalation.

While trifuhalol A proved its significant efficacy in controlling the allergic inflammatory cytokines from OVA inhalation exposure, the corticosteroid medication, dexamethasone treatment, continuously showed significant protection as well. On the other hand, the dexamethasone treatment showed the typical side effect of splenic atrophy, which was not found in the AC extract and trifuhalol A treatments (Figure S1). Particular interest exists in utilizing natural product-originated compounds, such as trifuhalol A, in combination with currently approved medications in asthma to observe the combination effect, which can lead to the decrease in known side effects while maintaining the therapeutic efficacy. Moreover, besides OVA, other allergens are used in mouse models of experimental allergic asthma. The house dust mite extract-induced model, for example, is known to have close correlation with clinical allergic asthma and may enhance our current findings when tested with AC and trifuhalol A in the future. Considering the reported side effects of corticosteroids and the therapeutic effect of trifuhalol A found in the current study, further research on the detailed mechanisms of action with trifuhalol A on various models of asthma will lead to the development of strong therapeutic candidates or combination therapeutics for allergic asthma.

## 5. Conclusions

Throughout the study, trifuhalol A, isolated from AC, exhibited the significant effect of reducing the Th2-specific cytokine release from the lung and airways to reduce the formation of allergen-specific IgE, which are involved in the suppression of eosinophils and mast cells activation, leading to the protection of the respiratory system from the structural changes found in allergic asthma. With the additional control of lung IL-1β, IL-6, and TNF-α levels after OVA inhalation exposure, trifuhalol A has strong potential to be further evaluated as a therapeutic candidate for allergic asthma.

## Data Availability

All data are available from the corresponding author upon request.

## Supplementary Materials

The following supporting information can be downloaded at https://www.mdpi.com/article/10.3390/cimb45110557/s1: Figure S1: Effect of dexamethasone, AC extract, and trifuhalol A on the weight of spleen after OVA inhalation exposure.
