# Supplementary material for: Anti-Allergic Inflammatory Effect of Agarum cribrosum and Its Phlorotannin Component, Trifuhalol A, against the Ovalbumin-Induced Allergic Asthma Model
Source: Curr Issues Mol Biol. 2023 Nov 5;45(11):8882–93. doi: 10.3390/cimb45110557 (PMC10669934; doi:10.3390/cimb45110557)
Supplement: Supplementary file 1 [file cimb-45-00557-s001.zip › cimb-2684166-supplementary.pdf]

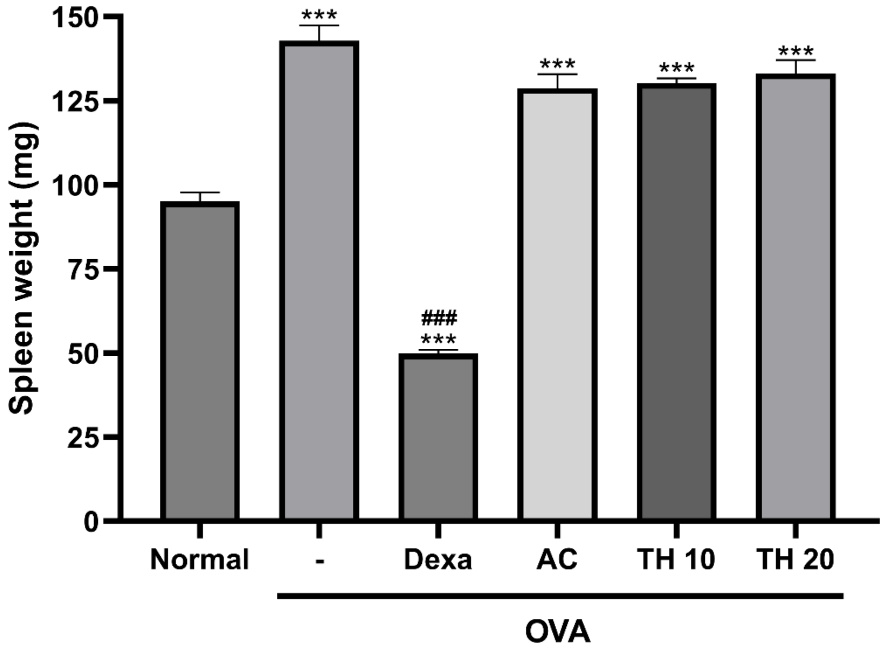

Figure S1. Effect of dexamethasone, AC extract, and trifuhalol A on the weight of spleen after OVA inhalation exposure. Average weight of harvested spleen is presented. Dexamethasone 1.5 mg/kg (i.p.), AC extract 100 mg/kg (p.o.), Trifuhalol A 10 and 20 mg/kg (p.o.) were treated for each group. Data are presented as mean ± S.E.M (N=5/group). *** Significantly different (p<0.001) from normal group. ### Significantly different (p<0.001) from OVA inhalation group.
